# Manifestations and Mechanism of SARS-CoV2 Mediated Cardiac Injury

**DOI:** 10.7150/ijbs.69677

**Published:** 2022-03-28

**Authors:** Si-chi Xu, Wei Wu, Shu-yang Zhang

**Affiliations:** Department of Cardiology, Peking Union Medical College Hospital, Chinese Academy of Medical Sciences and Peking Union Medical College, Beijing, China.

**Keywords:** COVID-19, SARS-CoV2, Heart, Cardiac injury, Myocarditis

## Abstract

Coronavirus disease 2019 (COVID-19), a global pandemic caused by severe acute respiratory syndrome coronavirus 2 (SARS-CoV2) had resulted in considerable morbidity and mortality. COVID-19 primarily posed a threat to the respiratory system and violated many different organs, including the heart, kidney, liver, and blood vessels with the development of the disease. Severe patients were often accompanied by cardiac injury, and once the heart gets damaged, the mortality of patients will significantly increase. The main clinical manifestations of cardiac injury range from myocarditis, heart failure (HF), arrhythmia, and Takotsubo cardiomyopathy (TCM). A high abundance of angiotensin-converting enzyme II (ACE2) on the membrane of cardiomyocytes makes it possible that the virus can directly attack cardiomyocytes as subsequently evidenced by the detection of spike protein and virus RNA in autopsy cardiac tissues. The secondary myocardial injury through systemic inflammatory and immune response also caused obvious cardiac damage. The pathological manifestations of heart tissue were diverse, varied from mild cardiomyocyte edema, myocardial hypertrophy, cardiomyocyte degeneration, and necrosis to severe myocarditis caused by lymphocyte and macrophage infiltration. However, the mechanism of heart injury was still unclear. Here, we summarized the clinical manifestations and mechanism of SARS-CoV2 mediated cardiac injury, providing a reference for cardiac treatment in critically ill patients.

## Introduction

The outbreak of COVID-19 in 2019 had spread almost the whole area of the world, which had done great harm not only to the public but also pose a great threat to global development. Although the average mortality of patients with COVID-19 was about 2%-3.4% 1-3, patients with COVID-19 who had developed severe pneumonia can rapidly aggravate into multi-organ failure and death within 2 weeks after dyspnea 4. COVID-19 had caused system inflammatory syndrome, and the cytokine/chemokines released into plasma could result in serious damage to other organs such as the heart, liver, kidney, and coagulation system. Multi-organ complications had accelerated the deterioration and there were no effective methods and pharmacological therapies to stop and cure this disease completely yet 5. Among the various complication of severe COVID-19, the cardiovascular symptom was the most significant and life-threatening. A high ratio of acute cardiac injury (19.7-33%), leading to significantly high mortality, had been reported in the former study 6. Patients with previous cardiovascular diseases (CVDs) may be faced with a greater risk of developing into severe conditions and comorbidities which also posed a large threat to the prognosis of the COVID-19 7. Troponin, a sensitive biomarker of cardiac injury, had been reported as a strong predictor of in-hospital mortality according to a previous systematic review. The mortality of patients with elevated troponin was five times higher than that of the normal group, with 55% sensitivity and 80% specificity 8. Successful treatment strategies for critical patients mainly depended largely on the maintenance of normal cardiac function. However, the specific mechanism of the heart was still unclear. Here we summarized the latest progress about the pathological manifestations of the heart by SARS-CoV2 and preliminary elaborate the underline mechanism.

## Basic annotation of cardiac injury

Recent studies on the COVID-19 pandemic found that cardiac injury occurred in up to 20-30% of patients and was associated with increased disease severity 9. The majority of patients with COVID-19 belong to the mild and moderate clinical symptoms range from slight palpitation, chest tightness, and wheezing at the early stage, but small part patients may rapidly progress into acute respiratory distress syndrome (ARDS) followed by multi-organ failure, causing a seriously high mortality rate. Our previous study demonstrated that patients with myocardial injury were older, had a higher prevalence of underlying CVDs, and in-Intensive Care Unit (ICU), CVDs complications, higher Acute Physiology and Chronic Health Evaluation II (APACHE- II) scores, and Sequential Organ Failure Assessment (SOFA) scores compared with non-myocardial injury patients. Myocardial injury on admission increased the risk of 28-day mortality, too 10.

## Manifestations of cardiac injury

### Myocarditis

Despite being a small part of virus-induced myocarditis, human coronaviruses had been proved to be correlated with myocarditis in patients of all different groups 11. The viral RNAs of Middle East respiratory syndrome coronavirus (MERS-CoV) and SARS-CoV, which belong to close relatives of SARS-CoV-2, were both detected in the heart tissues of infected animals, suggesting that these coronaviruses possess cardio tropism. Several COVID-19-related myocarditis cases had been reported. The most common presenting symptoms of COVID19 related myocarditis were fever (62%), shortness of breath (48%), cough (48%), and chest pain (34%). Other symptoms such as fatigue, nausea, vomiting, diarrhea, myalgia, weakness, and headache were also reported 12. Results of blood tests often showed elevated levels of lactate and other inflammatory markers, including C-reactive protein (CRP), erythrocyte sedimentation rate (ESR), and procalcitonin, which were usually raised by the clinical presentation. The cardiac inflammatory response caused by SARS-CoV-2 varies from person to person. Therefore, the diagnosis of myocarditis still largely depends on clinical symptoms, electrocardiogram, cardiac magnetic resonance (CMR), and even myocardial biopsy and pathology. The detection of virus in heart tissues has not always been associated with the occurrence of myocarditis. From one patient with endomyocardial biopsy (EMB) analysis, the SARS-CoV-2 was not identified in the myocardium, suggesting acute virus-negative lymphocytic myocarditis. Fulminant myocarditis was reported with increased septal thickness with normal left ventricular (LV) diastolic dimensions, possibly caused by myocardial edema, which was detected by CMR 13. No typical CMR manifestations accompanied with uncommon invasive procedure of EMB resulted in low detection of the pandemic of COVID-19 related myocarditis. It was critical to distinguish fulminant myocarditis from sepsis-induced systemic inflammatory response syndrome (SIRS) because fluid resuscitation, a common sepsis protocol, aggravated fulminant myocarditis with fluid overload. Baseline testing of cardiac enzymes (troponin and N-terminal pro-B-type natriuretic peptide [NT-proBNP]) on hospital admission was necessary for elevated troponin and NT-proBNP were observed in the COVID-19-related myocarditis cases.

### Arrhythmias and sudden cardiac arrest

Arrhythmia was recognized as one of the earliest clinical manifestations of COVID-19. An early study of 137 patients in Wuhan showed that 7.3% had regarded palpitations as one of their main presenting symptoms from the beginning of the disease 14. Arrhythmia caused by COVID-19 ranged from atrial arrhythmias, ventricular arrhythmias, and cardiac arrest. Tachyarrhythmia was commonly seen in patients with COVID-19, while bradyarrhythmia was infrequent and they seem to occur suddenly and most of these cases were due to high-level atrioventricular block. Our study demonstrated that the incidence of tachyarrhythmias in non-survivor was much higher than in survivors and the use of beta-blockers was associated with a lower risk of death 15. The abnormal electrocardiogram in electrocardiogram (ECG) findings were closely related to the underlying severity of COVID-19 patients. Patients with an elevated level of Troponin T (TnT) had a higher incidence of malignant arrhythmias such as haemo-dynamically unstable ventricular tachycardia or ventricular fibrillation than those with normal TnT levels 16. Another study demonstrated that patients with cardiac injury showed prolonged corrected QT interval (QTc) interval, more abnormal T wave leads, and more T wave alteration. In addition, the number of leads with abnormal T waves was positively associated with a high-sensitivity TnT (hs-cTnT) level 17. Another survey indicated ECG signs refer to acute right ventricular pressure overload (RVPO) were detected in 30% of the patients which may be caused by pulmonary hypertension or ARDS. The feature of pulmonary embolism was also displayed, about 43 (10%) patients had the S1Q3T3 pattern, 38 (9%) had incomplete right bundle branch block (RBBB), and 49 (11%) had complete RBBB. Non-specific repolarization abnormalities were 41% of 176 patients and low QRS voltage in peripheral leads was present 23 (5%) 18. Another study demonstrated that viral infection of human-induced pluripotent stem cell-derived cardiomyocytes (IPSC-CMs) progressively impairs both their electrophysiological and contractile function and result in widespread cell death. SARS-CoV-2 leads to reduced beating rate, lower depolarization spike amplitude, and decreased electrical conduction velocity. Overall, abnormalities in the generation and propagation of electrical signals were obvious even in the absence of extensive cell death, suggesting that SARS-CoV-2 infection in cardiomyocytes could directly lead to arrhythmia 19.

### Heart Failure (HF)

HF was common in cases with severe COVID-19. In an early study from Wuhan involving 799 patients, HF was one of the most observed complications of COVID-19, with a reported incidence of 24% in all patients and 49% of those patients died in the end 20. Acute HF was regarded to be a possible direct consequence of COVID-19, with a dramatic influence on mortality. An Italian multicenter study demonstrated that 9.1% of patients during hospitalization were aggravated into acute HF due to COVID-19 and almost half of them belong to “de-novo” HF without previous HF history 21. In another study that included 3080 COVID-19 patients in Madrid (Spain), about 2.5% of patients were accompanied with acute HF and resulting in higher mortality as much as 46.8% than that of 19.7% in patients without HF 22. LV diastolic abnormity with elevated filling pressures (E/e' ratio) was more commonly seen concerning systolic dysfunction in COVID-19 patients presenting acute HF. Consistently, patients admitted to the hospital showed a high likelihood of preserved ejection fraction (HFpEF) as compared with patients without COVID-19. As with left ventricular HF, the incidence of right ventricular HF in COVID-19 secondary to ARDS and pulmonary hypertension was higher in ICU patients. In a prospective echocardiography examination of 100 COVID-19 patients, 69% showed signs of HF, while 39% had right ventricular (RV) dilation or dysfunction, 16% had LV diastolic dysfunction, and 10% had LV systolic dysfunction 23. Various second factors such as pulmonary vasoconstriction, ARDS, mechanical ventilation, and extracorporeal membrane oxygenation (ECMO) related to serious virus infection may easily lead to volume and pressure afterload of RV. Our echocardiographic of 43 patients with COVID-19 revealed high frequencies of pericardial effusion, increased left ventricular mass index, elevated relative wall thickness, and reduced left ventricular stroke volume index (LVSVi) and cardiac index. Moreover, cardiac index was the strongest predictor of in-ICU death. The swollen heart had accelerated the progress of HF 24.

### Acute coronary syndrome (ACS)

The diagnosis ratio of ACS was lower during the COVID-19 pandemic owing to the particularity of the disease and lack of an efficient diagnostic process. Hospitalization rates of acute myocardial infarction (MI) and duration of symptom onset to first medical contact were significantly lower in Turkey during the COVID-19 outbreak. And those patients with high thrombus grades were more commonly seen and had higher mortality rates during the break 25. From the perspective of clinical founding, SARS-CoV-2 had also been associated with serious ACS, especially in those with basic CVDs. The ACS of COVID-19 related myocardial injury mainly belonged to type 2 MI due to a mismatch in myocardial oxygen supply and demand. Severe respiratory viral infection leads to the unbalance of oxygen supply and demand, which was initiated by hypoxemia and vasoconstriction, as well as an inflammatory response 26. Patients who suffer from type II MI usually do not have angina, a mild increase of troponin, BNP, and without obvious ischemia. However, the systemic inflammatory response can also increase the potential of plaque rupture and acute thrombus formation, resulting in type I MI. One clinical study recruited 18 confirmed COVID-19 with ST-segment elevation on electrocardiography demonstrated that about 6 of 9 patients (67%) had obstructive disease by coronary angiography 27. It was essential to recognize ACS and ACS-mimicker to provide proper treatment and avoid additional risks. Anticoagulation and antiplatelet therapy increase the risk of bleeding from myocarditis and cardiomyopathy while delayed vascular recanalization will lead to more cardiomyocyte necrosis and cause heart pump failure.

### Takotsubo cardiomyopathy (TCM)

Scattered cases of TCM were reported as a complication of COVID-19, about 90% of the cases occurred in women with a mean age of 64.6 years. TCM was usually more prevalent among postmenopausal women, but it was observed that both genders were equally affected in one of the previous studies 28. The specific reason for TCM may be relevant to plasma levels of catecholamines (epinephrine, norepinephrine, and dopamine) and simultaneously overactivated sympathetic system. The toxicity of catecholamine accompanied with waterfall-released inflammation and cytokine storm may result in the incidence of TCM. According to a recent survey, the incidence of TCM had increased in the COVID-19 pandemic compared to pre-pandemic (7-8% vs 1-2%) 29. From a retrospective study conducted by Shruti Hegde, who had discovered that COVID-19 complicated by TCM had a high mortality rate 30. TCM may also last for a long time after recovery COVID-19 infection as a delayed report of 78% recovered COVID-19 patients had positive cardiac MRI findings 31.

### Kawasaki-like disease

Kawasaki disease (KD) is referred to as an acute systemic inflammatory disease of medium and small-sized vessels that mostly involve children under five years old. A recent report by Verdoni found that 30-fold increased incidence of Kawasaki-like disease over a month shortly after the spread of SARS-CoV-2 in Bergamo-Italy and the severity of KD also increased during this period 32. According to a retrospective survey conducted in Paris, Kawa-COVID-19 likely represented a new systemic inflammatory syndrome temporally associated with SARS-CoV-2 infection in children manifested with older age, lower platelet count, a higher rate of myocarditis, and resistance to first intravenous immunoglobulin (IVIG) treatment. The latest observational study found that 76% of the 21 children and adolescents with Kawasaki-like disease had myocarditis diagnosed by cardiac troponins, ECG, and echocardiogram 33. A post-infectious process with delayed immune activation leading to a cytokine burst, responsible for fever, skin rash, HF, and a major inflammatory syndrome, seems likely the presumed pathophysiology of 'classical' KD 34.

## Pathological manifestations of heart injury

**Figure 1:**
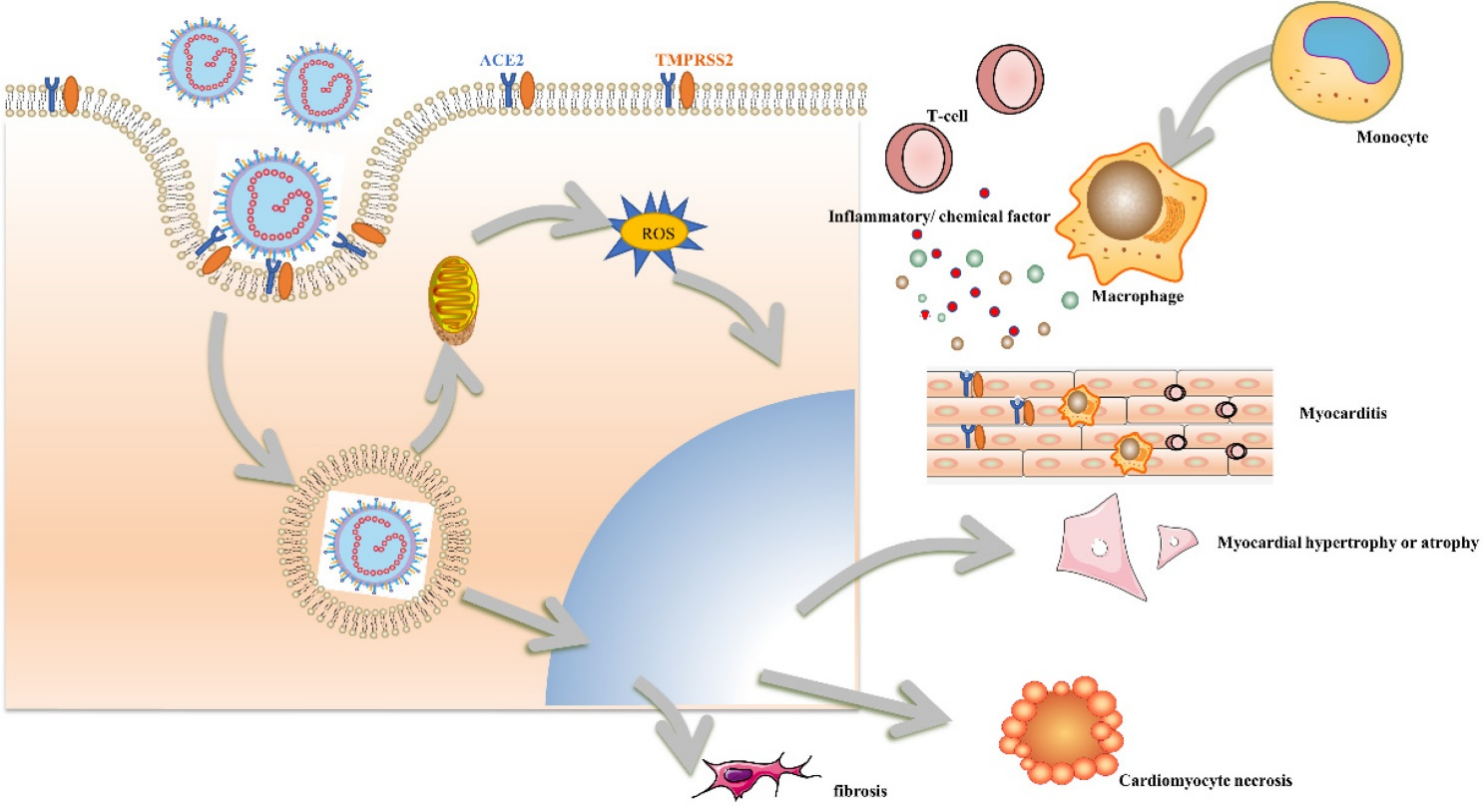
Changes of cardiomyocytes induced by SARS-CoV2.

The pathology manifestations of the heart ranged widely from no obvious histological changes to diffuse cardiac injury. An early autopsy of a patient with COVID-19 and ARDS who died of a sudden cardiac arrest displayed no evidence of obvious myocardial structural involvement 35. Another autopsy study demonstrated that the virus was not located in the cardiomyocytes but scattered in the interstitial cells or macrophages, which was inconsistent with the clinical symptom of fulminant myocarditis 36. According to our recent autopsy report from 26 patients with COVID-19, almost all the major organs could be the target of the virus. SARS-CoV2 was detected in the heart of 5 patients as long as 16-65 days after the symptom onset 37. Zsuzsanna Varga found one patient with an accumulation of inflammatory cells around the endothelium, as well as apoptotic bodies in the heart 38. Another previous pathology study had reported active lymphocytic endotheliitis and endothelial cells containing coronavirus-like particles, which claims the microvascular direct damage 39. Only one study was carried out in patients without previous cardiovascular disease or any clinical signs of cardiac involvement. Though SARS-CoV-2 in cardiomyocyte was all invariably detected, a variable response of cardiomyocyte injury was observed, ranging from absence of cell death, subcellular alterations to intracellular edema, and abnormal, disrupted, and clumped myofibrils, and no significant large-area cell death 40. Representative cardiac autopsy and manifestations were summarized as follows in Table 1 and Figure 1.

## The main mechanism mediating cardiac injury

### Virus directly induced cardiac toxicity by ACE2

Early studies had reported cases of acute COVID-19 associated myopathy without prior CVDs, indicating that SARS-CoV-2 may directly induce cardiac damage. Previous murine models and human autopsy had proved that SARS-CoV can down-regulate ACE2 in myocardial and pulmonary and thereby lead to myocardial inflammation, lung edema, and acute respiratory failure 58. SARS-CoV-2 may share the same mechanism with SARS-CoV due to the highly homologous genome of the two viruses. Another hypothesis also had a suspicion that people with pre-exit CVDs had a higher risk of infection and poor outcomes by the higher expression of ACE2. The latest report detected the soluble level of ACE2 (sACE2) and discovered higher levels of sACE2 were associated with male, sex, CVDs, diabetes, and older age. Moreover, the sACE2 level was most associated with the level of cardiac injury biomarker of NT-proBNP, and hs-cTnT. Recent reports had proved that IPSC-CMs were susceptible to SARS-CoV-2 infection. And they demonstrated that SARS-CoV-2 can enter IPSC-CMs via ACE2 by microscopy. SARS-CoV-2 infection activated innate immune response and antiviral clearance gene pathways while inhibiting metabolic pathways and suppressing ACE2 expression 59. SARS-CoV-2 had 10 to 20-fold greater binding affinity than that of SARS-CoV which makes it novel and strong infectious ability 60. ACE2 transforms angiotensin (Ang) I and Ang II into Ang 1-9 and Ang 1-7 respectively to regulate the balance of the cardiovascular, renal, and pulmonary systems. Downregulation of ACE2 due to viral blockade accompanied with unchanged ACE result in accumulation of Ang II. Ang II interacts with Ang II type 1 receptor (AT1R) and trigger the activation of proinflammatory and profibrotic cascades. Therefore, COVID-19-induced Ang II upregulation by incompatible ACE/ACE2 may result in local inflammation, capillary leakage, a procoagulant state, mitochondrial oxidative damage, and cardiomyocyte apoptosis 61.

### Inflammation and immune response

Immune organs were the second most affected system by SARS-CoV2. Pathological investigations of COVID-19 had discovered splenic atrophy, with an obvious reduction in the number of lymphocytes and neutrophils, as well as necrosis and hemorrhages. Country to lymphopenia in peripheral blood observed in severe cases, an increase in systemic inflammatory factors had been observed in subjects with COVID-19, which corresponds to the characteristics of a cytokine release syndrome (CRS) 62. Our study indicated that plasma levels of C-reactive protein (CRP), interleukin-2 receptor (IL-2R), IL-6, IL-8, and tumor necrosis factor-α (TNF-α) were statistically higher in the myocardial injury group than in the non-myocardial injury group and CRP, as well as TNF-α, were positively correlated with the incidence of myocardial injury 63. Moreover, over-activated systemic inflammation secondary to the infection may lead to an increase in metabolic demand, which may potentially aggravate coronary plaque ruptures and enhance the ratio of stent thrombosis. The immune disorder was also proved by our early study that antiphospholipid antibodies (aPLs) were commonly seen in critically ill patients and multiple medium or high levels aPLs may help identify patients at risk of developing cerebral infarction 64. In addition to lymphocyte, macrophage infiltration was also commonly seen in the autopsy reports. RNA-seq (RNA-sequencing) from autopsy heart samples and non-COVID-19 donors discovered the significant upregulation of C-C motif chemokine ligand 2 (CCL2), a well-known chemoattractant for migrating macrophages. Macrophages secreted IL-6 and TNF-α when exposed to SARS-CoV-2 in coculture platform containing IPSC-CMs and macrophages, which lead to reactive oxidative species (ROS) production and apoptosis of cardiomyocytes 65. Compared with lymphocyte-associated inflammation, macrophage-associated inflammatory response was usually more serious, and the clinical symptom was more obvious. Patients with mild cardiac injury had scattered CD4 and CD8 lymphocytes and myocyte necrosis, while patients with more serious clinical presentations, such as fulminant myocarditis, usually had CD68+ macrophages infiltration and foci of cardiomyocyte necrosis. Macrophage infiltration was more commonly seen in the heart and correlate to an elevated systemic level of proinflammatory cytokines response. SARS-CoV2 infection upregulates several inflammation-related genes, including the proinflammatory TNF-α in IPSC-CMs. The pretreatment of IPSC-CMs with TNF-α significantly increased the expression of ACE2 and transmembrane serine protease 2 (TMPRSS2), SASR-CoV2 entry receptors. The TNF-α pretreatment enhanced the entry of GFP-expressing SARS-CoV2 pseudovirus into IPSC-CMs, and the neutralization of TNF-α slow down the TNF-α-enhanced viral entry. Therefore, SARS-CoV2 can stimulate higher TNF-α expression, which in turn enhanced the SARS-CoV2 viral entry 66. Immunomodulatory therapy had been investigated to modulate immune hyperactivity and the cytokine storm that occurs in the later stage of COVID-19 infection. Monoclonal antibodies had shown some promise in early studies. IL-6 inhibitors tocilizumab and sarilumab had shown early benefit and were being studied in ongoing randomized trials 67.

### Mitochondrial dysfunction and oxidative stress

SARS-CoV-2 infection was associated with unbalanced mitochondrial dynamics followed by consequent oxidative stress, pro-inflammatory state, cytokine production, and cell death. SARS-CoV-2 altered mitochondrial dynamics at different levels including mitochondrial DNA damage, changes in mitochondrial membrane potential, calcium homeostasis, and production of ROS during the infection. Exogenous chemical factors and endogenous cytokines can also do damage to mitochondria and cause mitochondrial dysfunction. When SARS-CoV-2 binds with ACE2 and entered into the plasma by endocytosis, the over- duplicate of DNA will trigger the disorder of energy metabolism which disturbed the balance of mitochondria‐endoplasmic reticulum and Golgi apparatus. In addition, the reduction of ACE2 will increase ROS generation due to enhanced NADPH activity and reduce mitochondrial homeostasis. A recent study of genome-wide transcriptional alterations induced by SARS-CoV-2 infection in IPSC-CMs had revealed that genes involved in mitochondrial function and energy production were downregulated, indicating that SARS-CoV-2 might promote a shift toward a glycolytic metabolism by suppressing mitochondrial oxidative phosphorylation, which could also favor its replication 68. Owing to the excessive production of ROS in the heart, a potential anti-oxidative therapy had been suggested to alleviate cardiogenic casualties caused by COVID-19. Potential antioxidants such as vitamin C (ascorbic acid) and vitamin E had been proposed because the reductive hydrogen atoms can react with ROS and then produce nontoxic water. Plant-derived molecules such as curcumin and baicalin may also have potential anti-oxidative efficacy, too 69. The previous studies illustrate that the antioxidant effects of vitamin D may be useful in the prevention and attenuation of COVID-19 symptoms and that a lower vitamin D status would confer susceptibility to morbidity and mortality by SARS-CoV-2 70.

### Endothelial cell (ECs) injury

Direct virus-related endothelial injury, hyper-coagulation state leading to occlusion, and microthrombi formation were commonly seen in autopsy and histological analysis. Another recent study revealed high expression of ACE2 on cardiac pericytes may also serve as the target of SARS-CoV-2 71. Despite direct endothelial cell injury, inflammatory infiltration into ECs promotes endothelialitis, perturbing ECs membrane function, loosening inter-endothelial junctions, and causing cell swelling even death. Activated endothelial cells become a source of proinflammatory and pathological ECs enhance ROS production, decrease vasodilative function, and were in favor of thrombus formation. Moreover, ECs increased their production of plasminogen activator inhibitor 1 (PAI-1) after SARS-CoV-2 infection which inhibited the conversion of plasminogen to plasmin and the degradation of clots and maintained a hypofibrinolytic state. Constantly activation of coagulation pathway with the potential development of disseminated or diffuse intravascular coagulation (DIC) was a general characteristic of severe infections with COVID-19, which can be reflected by the increase of fibrin degradation fragments (D-dimer). Moreover, abnormal ECs failed to release enough NO resulting in obvious vessel constriction. NO deficiency results in vascular smooth muscle contraction, impeding the ability to neutralize ROS and NO-related antiviral capability. As the direct target of SARS-CoV-2, early intervention on vascular endothelium may block the spread of the virus and reduce thrombosis incidence. Active use of prophylactic or even therapeutic anti-coagulation therapies was widely recommended during the treatment. Tang et al. demonstrated that prophylactic anticoagulant therapy was associated with a decreased mortality in COVID-19 patients 72.

### Hypoxemia

The cardinal reason for hospital admission in COVID-19 was hypoxemia. Even younger patients with no prior history of lung disease can have severe pneumonia and require invasive ventilation. Constant virus replication in type 2 alveolar epithelial cells results in cell death, and the release of lysate fragments leads to the infiltration of inflammatory cells, increased secretion of airway mucus, infiltration of monocyte macrophages. These substances accumulating in the airway greatly increase airway obstruction and reduce alveolar ventilation. Moreover, the hyaline membranes and widened alveolar walls also inhibit oxygen diffusion and aggravate the retention of carbon dioxide. Except for the reduction of respiratory oxygen supply, edema of cardiomyocytes, infiltration of inflammatory cells and fibrosis also aggravate the disorder of oxygen diffusion. At the same time, extensive microvascular thrombosis further aggravates the imbalance of oxygen supply to the whole heart. The degree of hypoxia in the cardiovascular system during COVID-19 can be reflected by the following indicators according to the previous study 73: 1. Continuously increasing and worsening acidemia or plasma lactate; 2. Increased requirements for a vasoactive agent to maintain normal blood pressure despite enough fluid resuscitation; 3. Increased alteration in blood pressure to changes in body position and intolerance of head-up positions; 4. Abnormal electrocardiogram and arrhythmias changes such as bradycardia and increased heart rate variability; 5. Abnormal cardiac function and elevated markers of myocardial injury: increased troponin levels or ultrasound evidence of impaired myocardial contractility. Hypoxia increased the incidence of ischemic heart disease, which lead to increased cardiac mortality.

### Stress state and unbalanced autonomic nervous

Stress had participated in progress in the COVID-19 related cardiac injury. Patients with COVID-19 result in a marked and appropriate acute cortisol stress response than in individuals without COVID-19. Furthermore, it was also believed that high cortisol concentrations were associated with increased mortality, reduced median survival, and bad prognosis 74. Over-accumulated glucocorticoid in the heart will reduce the velocity of relaxation in the papillary muscle and shorten the total relaxation time without a significant effect on contraction time 75. The stress state was often accompanied by dysregulation of the autonomic nervous which was mainly displayed with overactivity of the sympathetic nerve. Cardiovascular complications such as arrhythmias, myocarditis, HF, and myocardial infarction frequently occurred during the process. All these conditions were negatively regulated by sympathetic overactivation and could reversely aggravate COVID-19 morbidity and mortality.

### Psychosocial disorder

The pandemic of COVID-19 associated with physical isolation practices was likely to result in a wide range of mental health and psychosocial disorder. Regardless of an increasing incidence of loneliness, anxiety, depression, suicidal accident, and post-traumatic stress, the pandemic may also increase social disharmony in restricted freedom, domestic violence, and relationship division. The pandemic and physical isolation had the potential to produce a vast array of mental health and psychosocial challenges both chronic and acute CVDs. The strategy of social distance and quarantine lead to global disengagement with the health care system. For example, the number of patients who had undergone cardiology procedures was all reduced over the post-COVID-19 outbreak. Patients who developed myocardial injury seem to be more than 10-times the possibility to be admitted to ICU or cardiac care unit, which may aggravate the deterioration of the disease by fear, anxiety, vulnerability, helplessness, and trepidation. Pessimism and loneliness had been proved to be associated with the development and progression of atherosclerosis with elevating peripheral vascular resistance and increased blood pressure 76. Conversely, dense living environments or proximity to family members over a prolonged period during the isolation period may inhibit independence and self-reliance or, in some cases, incite conflict and intimate partner violence (IPV). The IPV often accompanied maladaptive or dysfunctional relationships with both elevating the risk of CVDs 77. Divorced or separated men had a higher risk of CVDs mortality, while women, especially those aged 55 years or older had a higher opportunity to develop TCM when confronted with extreme distress in life.

### Drug-induced cardiotoxicity

Due to the rapid spread and high transmission of COVID-19, many pharmaceutical drugs for the treatment and prevention of COVID-19 had been applied in clinical treatment. Cautious should be paid for the off-label use of previously approved drugs due to cardiac toxicity especially, in severe cases with multiple comorbidities. Long QT was commonly seen in anti-viral therapies such as hydroxychloroquine. Hydroxychloroquine can block voltage-gated potassium channels (Kv11.1) and induce drug-induced QT prolongation by prolonging action potential duration, thereby increasing the risk of drug-induced torsades de pointes (DI-TdP) and sudden cardiac death. Tocilizumab, a monoclonal antibody acting as an IL-6 inhibitor, currently tested in COVID-19 patients, had been reported to shorten QT interval. Ritonavir inhibited cytochromeP4502D6 (CYP2D6) and induced CYP2B6, CYP2C19, CYP2C9, and CYP1A2 which disturbed the metabolism disorder and laid a solid foundation of the drug's adverse effect on the heart under normal dosage. The main adverse reactions of IFN- α include ischemic cardiomyopathy, arrhythmia, hypertension, or hypotension 78. The common adverse reactions of ribavirin were cardiotoxicity, dyspnea, and chest pain, especially in patients with chronic obstructive pulmonary disease and bronchial asthma. Abidol can mainly cause bradycardia. Another special cardiotoxic associated drug was vaccines. Myocarditis had been considered as a rare complication (around 1/100 000 in the general population) of COVID-19 mRNA vaccinations, especially in young adult and adolescent males. Myocarditis usually occurs 2 to 3 days after the second dose of mRNA vaccination, and almost all people show chest pain. Most electrocardiograms showed ST-segment elevation, and CMR also had corresponding abnormal changes. Myocarditis was more common in male patients may be associated with sex hormone differences in the immune response. However, it is gratifying that most patients will return to normal with and without special intervention 79.

## Further advice for research and clinical practice

Subsequent studies can accurately focus on the specific molecular mechanism and related proteins of cardiac injury. How do T lymphocyte cells and macrophages participate in cardiac injury? Humanized cells and even cardiac organoids can be widely used to explore the characteristics of COVID-19 mediated cardiac damage and screen relevant antiviral drugs to delay and reduce disease development. In clinical practice, we should be alert to high-risk groups, especially those elderly patients with basic CVDs. Once a patient had arrhythmias or cardiac symptoms that cannot be routinely explained, COVID-19 related cardiac injury should be considered at first. Troponin can be used as a sensitive index to evaluate heart injury. Early evaluation of cardiac function in high-risk patients was essential for subsequent evaluation and judgment. Patients with severe myocarditis should be differentiated from sepsis-related cardiac injury to facilitate the second treatment. At the same time, the risk of thrombosis should be assessed for each patient as soon as possible and add anticoagulants when necessary.

## Conclusion

SARS-CoV2 can directly attack the heart via ACE2, causing cardiomyocyte edema, degeneration, and necrosis. At the same time, the immune response induced by the virus resultsed in inflammatory associated myocarditis, activation of the body's sympathetic adrenal medullary system, increased oxygen consumption, which disturbed the imbalance of cardiac oxygen supply resulting in plaque rupture and MI. Moreover, the in-completed vascular endothelium leads to vasoconstriction, micro-thrombosis, aggravated the disorder of cardiac energy metabolism, leads to oxidative stress and the production of ROS. IPSC, a proper method to simulate human-derived cardiomyocytes infected with SARS-CoV2 reveals the mechanism of myocardial cell injury and provided reliable evidence for the protection of the heart in subsequent critically ill patients. However, the response of heart damage triggered by COVID-19 was perplexing and varied from person to person. Therefore, it was urgent to explore further study in the whole body to reveal heart damage. At present, due to the limit of animal experiments, there was still a lack of research on COVID-19 mediated cardiac injury in vivo, and a variety of composite mechanisms still need to be explored.

## Figures and Tables

**Table 1:** Pathology results of cardiac autopsy specimens in patients with COVID-19.

Name	Number	Cardiac pathology(number)	Virus(number)
Yao et al. 35	1	NM	Negative (1)
Lindner et al. 36	39	Mononuclear cells infiltration	Positive (24)
Yao et al. 37	26	NM	Positive (5)
Varga et al. 38	3	Lymphocytic endotheliitis	Positive (1)
		No sign of lymphocytic myocarditis.	
Fox et al. 39	22	Scattered myocyte necrosis	Positive(endothelial)
		Severe right ventricular dilatation	Negative(cardiomyocyte)
		Endothelial cell swelling	
		CD4 and CD8 lymphocytes near vascular	
Bulfamante et al. 40	6	Inflammatory infiltrates	Positive (6)
		Interstitial macrophages infiltration	
		Cardiac edema,	
		Damaged sarcomeres	
		Disrupted and clumped myofilaments	
		No endothelial viral cytopathic effects	
		No endotheliitis	
Hanley et al. 41	9	Pericarditis (2)	Positive (2)
		Thrombotic (5)	
		Marantic Endocarditis (1)	
		Left ventricular hypertrophy (4)	
		Cardiac amyloidosis (1)	
Yao et al. 42	3	Cardiomyocyte hypertrophy	Negative (3)
		Degeneration/Necrosis edema	
		Lymphocyte, monocytes, and neutrophils and CD4 positive T cells	
		Swelling and dissolution of myocardial fibers	
Wang et al. 43	2	Myocardial degeneration	Negative (2)
		Myocardial atrophy	
		Interstitial fibrous tissue hyperplasia	
		CD20-positive B cells and CD3-positive T cells scattered in the heart	
Tian et al. 44	2	Edema/Fibrosis	Positive (2)
		Myocardial hypertrophy	
		No inflammatory cellular infiltration	
Dolhnikoff et al. 45	1	Thickened endocardium	Positive (1)
		Thickened myocardium	
		Myocarditis/Pericarditis/Endocarditis	
		CD68+ macrophages	
		CD45+ lymphocytes	
Wichmann et al. 46	12	Cardiac hypertrophy (8)	Positive (5)
		Coronary artery sclerosis (9)	
Lax et al. 47	11	Cardiac hypertrophy (11)	NM
		Fibrosis (10)	
		Amyloidosis (1)	
		Thrombosis (1)	
		Lymphocyte infiltration (1)	
Buja et al. 48	23	Cardiomegaly (13)	NM
		Cardiomyocyte injury (8)	
		Lymphocytic epicarditis/pericarditis (3)	
		Lymphocytic myocarditis (1)	
Fox et al. 49	9	Cardiomegaly	NM
		Extreme right ventricular dilatation	
		Scattered myocyte necrosis	
Falasca et al. 50	22	Myocarditis (12)	NM
		Vasculitis (8)	
		Inflammatory infiltrate (16)	
		Focal necrosis (8)	
		Pericarditis (13)	
		Vascular fibrosis (6)	
		Hemorrhage	
Bradley et al. 51	14	Fibrosis (14)	Positive (2)
		Myocyte hypertrophy (13)	
		Lymphocytes around necrotic myocytes (1)	
		Myocardial amyloid (1)	
Basso et al. 52	21	Lymphocytic myocarditis (3)	NM
		Macrophage infiltration (18)	
		Pericarditis (4)	
		Small vessel microthrombi (4)	
Menter et al. 53	21	Myocardial hypertrophy (15)	NM
		Senile amyloidosis (6)	
		Myocardial cell necrosis (3)	
		Acute myocardial infarction (1)	
Pellegrini et al. 54	40	Myocyte necrosis (14)	Positive (cardiomyocyte 3)
		Epicardial coronary artery thrombus (3)	
		Microthrombi (9)	Negative (endothelial 5)
		Hypertrophy of myocardium (29)	
		Amyloidosis (6)	
Gauchotte et al. 55	1	Macrophages and CD8+ cytotoxic T infiltration	Positive
Xu et al. 56	1	Interstitial mononuclear inflammatory infiltration	NM
Weckbach et al. 57	5	Macrophage infiltration (5)	Negative (5)
		Lymphocytic myocarditis (1)	

NM-not mention.

## Abbreviations

SARS-CoV2: severe acute respiratory syndrome coronavirus 2
HF: heart failure
TCM: Takotsubo cardiomyopathy
ACE2: angiotensin-converting enzyme II
CVDs: cardiovascular diseases
ARDS: acute respiratory distress syndrome
ICU: intensive Care Unit
APACHE-II: Acute Physiology and Chronic Health Evaluation II
SOFA: Sequential Organ Failure Assessment
MERS-CoV: Middle East respiratory syndrome coronavirus
CRP: C-reactive protein
ESR: erythrocyte sedimentation rate
CMR: cardiac magnetic resonance
EMB: endomyocardial biopsy
LV: left ventricular
SIRS: sepsis-induced systemic inflammatory response syndrome
NT-proBNP: N-terminal pro-B-type natriuretic peptide
ECG: electrocardiogram
TnT: troponin T
QTc: corrected QT interval
hs-cTnT: high-sensitivity TnT
RVPO: right ventricular pressure overload
RBBB: right bundle branch block
IPSC-CMs: human-induced pluripotent stem cell-derived cardiomyocytes
HFpEF: preserved ejection fraction
RV: right ventricular
ECMO: extracorporeal Membrane Oxygenation
LVSVi: left ventricular stroke volume index
ACS: acute coronary syndrome
MI: myocardial infarction
KD: Kawasaki disease
IVIG: intravenous immunoglobulin
Ang: angiotensin
AT1R: ang II type 1 receptor
CRS: cytokine release syndrome
CRP: C-reactive protein
IL: interleukin
IL-2R: interleukin-2 receptor
TNF-α: tumor necrosis factor-α
APLs: antiphospholipid antibodies
RNA-seq: RNA-sequencing
ROS: reactive oxidative species
DIC: disseminated or diffuse intravascular coagulation
ECs: endothelial cells
IPV: intimate partner violence
DI-TdP: drug-induced torsades de pointes
CCL2: C-C motif chemokine ligand 2
